# Evaluating the effectiveness of different perceptual training methods in a difficult visual discrimination task with ultrasound images

**DOI:** 10.1186/s41235-023-00467-0

**Published:** 2023-03-20

**Authors:** Jessica E. Marris, Andrew Perfors, David Mitchell, Wayland Wang, Mark W. McCusker, Timothy John Haynes Lovell, Robert N. Gibson, Frank Gaillard, Piers D. L. Howe

**Affiliations:** 1 Melbourne School of Psychological Sciences, University of Melbourne, Parkville, Australia; 2 Radiology, Sligo University Hospital, Sligo, Ireland; 3 Department of Radiology, The Royal Melbourne Hospital, Parkville, Australia; 4 Department of Radiology, University of Melbourne, Parkville, Australia

**Keywords:** Perceptual learning, Perceptual training, Perceptual expertise, Radiology

## Abstract

Recent work has shown that perceptual training can be used to improve the performance of novices in real-world visual classification tasks with medical images, but it is unclear which perceptual training methods are the most effective, especially for difficult medical image discrimination tasks. We investigated several different perceptual training methods with medically naïve participants in a difficult radiology task: identifying the degree of hepatic steatosis (fatty infiltration of the liver) in liver ultrasound images. In Experiment 1a (*N* = 90), participants completed four sessions of standard perceptual training, and participants in Experiment 1b (*N* = 71) completed four sessions of comparison training. There was a significant post-training improvement for both types of training, although performance was better when the trained task aligned with the task participants were tested on. In both experiments, performance initially improves rapidly, with learning becoming more gradual after the first training session. In Experiment 2 (*N* = 200), we explored the hypothesis that performance could be improved by combining perceptual training with explicit annotated feedback presented in a stepwise fashion. Although participants improved in all training conditions, performance was similar regardless of whether participants were given annotations, or underwent training in a stepwise fashion, both, or neither. Overall, we found that perceptual training can rapidly improve performance on a difficult radiology task, albeit not to a comparable level as expert performance, and that similar levels of performance were achieved across the perceptual training paradigms we compared.

**Supplementary Information:**

The online version contains supplementary material available at 10.1186/s41235-023-00467-0.

## Introduction

With practice and experience, humans learn to extract the relevant perceptual features that guide decisions about stimuli in their environment, even when these features are difficult to verbalise (Kellman & Garrigan, 2009). Training that aims to improve perceptual skills is referred to as *perceptual training* (Chen et al., 2017), whereas the term *perceptual learning* describes the improvement in task performance (e.g. the ability to identify, detect, and discriminate stimuli) that results from this training (Sagi, 2011). Perceptual learning occurs across a wide range of simple visual tasks with basic stimuli (e.g. dots, line segments, and Gabor patches), such as motion direction detection (Ball & Sekuler, 1987), orientation discrimination (Fiorentini & Berardi, 1980), and texture discrimination (Karni & Sagi, 1991).

Perceptual training techniques have been increasingly applied to real-world visual tasks with complex stimuli. A growing body of work in the medical domain—for example, in radiology (Chen et al., 2017; Frank et al., 2020; Johnston et al., 2020; Sha et al., 2020; Sowden et al., 2000), dermatology (Rimoin et al., 2015; Xu et al., 2016), histopathology (Krasne et al., 2013), and cytopathology (Evered et al., 2014)—has found that perceptual training can lead to rapid and substantial improvements in performance on visual tasks with medical images. These findings are particularly relevant because medical professionals, such as radiologists, undergo many years of training to develop the expertise to interpret complex medical images. Traditionally, radiologists are trained to interpret and diagnose medical images in a primarily rule-based fashion, although this may not be the most efficient approach for learning complex visual tasks that require perceptual decisions (Johnston et al., 2020). The findings from the perceptual training literature suggest that perceptual training techniques could usefully supplement the traditional training that radiologists receive. Perceptual training also offers the benefit of immediate feedback, which has been found to be essential for learning to interpret radiology images (Sha et al., 2020), and is often delayed or absent in real-world medical image training.

However, the extent to which perceptual training is beneficial remains somewhat unclear: is it possible for participants to reach (or at least approach) expert-level performance when the tasks are highly complex? Whilst many studies have shown that perceptual training can lead to improvements in performance in visual tasks in the medical domain, relatively few studies have explored whether it is possible for participants to achieve similar levels of performance to experts, and if so, when. One exception is a study by Chen et al. (2017), who compared the performance of medically naïve participants that underwent perceptual training to identify proximal neck of femur fractures in X-ray images to that of experts (board-certified radiologists and radiology residents) across a series of experiments. The mean accuracy of the participants was approximately 90% after only two perceptual training sessions, which was only slightly lower than the accuracy of experts (94%). Whilst pre-training accuracy was not assessed in this experiment, Chen et al. (2017) found that pre-training accuracy was only slightly above chance (55.9%) in two similar experiments. This finding suggests that perceptual training can be a practical and efficient way of obtaining medical image discrimination expertise.

Given its potential usefulness, it seems timely to ask if perceptual training techniques are effective for medical image discrimination tasks that require finer judgements (i.e. beyond a two-choice judgement). Additionally, with such tasks of increased difficulty, is there a particular perceptual training technique that is more effective? The most common and simple perceptual training technique is to present stimuli sequentially. On each training trial, participants make a judgement (e.g. “Is there a hip fracture present?”) about a single stimulus and are then informed if they were correct. Although similar techniques are used in the categorisation literature (category learning and perceptual learning likely result from overlapping mechanisms; Carvalho & Goldstone, 2016), we refer to this as *standard perceptual training* as our review focuses on the perceptual training literature. However, recent successes with alternative training techniques—for example, training participants with comparison images (e.g. Sha et al., 2020) or supplementing standard perceptual training with annotated feedback (e.g. Chen et al., 2017; Frank et al., 2020; Johnston et al., 2020)—question whether the standard perceptual training technique is the most effective, especially for more challenging perceptual tasks than what have typically been studied (i.e. beyond two-choice tasks).

Our overarching aim is to assess which perceptual training methods are the most effective for training medically naïve participants to improve their performance in a difficult real-world medical image discrimination task. To address this goal, we systematically tested different perceptual training procedures across a series of experiments. In our studies, we chose to assess perceptual training with a task which experts and trainee radiologists find difficult: identifying the degree of *hepatic steatosis* (fatty infiltration of the liver) on ultrasound images. Additionally, we sought to gain a better understanding of the limits of these perceptual training techniques in our task, by comparing the post-training performance of trained novices to an estimate of expert performance.

## Experiment 1a

Traditionally, perceptual learning studies with simple stimuli and tasks have involved multiple sessions with thousands of trials (Dosher & Lu, 2017; Gauthier et al., 1998). However, studies with complex real-world images tend to involve substantially fewer sessions and trials. This is often due to practical constraints such as the limited availability of suitable images (Chen et al., 2017) and time constraints related to recruiting and maintaining participants. Despite a shorter amount of perceptual training, many of these studies have found significant performance improvements (e.g. Chen et al., 2017; Johnston et al., 2020; Sha et al., 2020), suggesting that perceptual learning with complex medical image discrimination tasks can occur rapidly. For instance, the top five performers in Chen et al.’s (2017) study could be trained up to a level approaching that of experts within an hour of training. These findings suggest that perceptual training can be efficient and effective, although the task employed by Chen et al. (2017) was very simple, requiring participants only to learn to make a binary judgement. It is therefore unclear to what extent perceptual training can be used to assist participants in learning a more difficult visual image classification task, especially one requiring more than binary judgements.

If we can train naïve participants to perform a difficult visual discrimination task at a level comparable to experts in a short period of time with standard perceptual training, then there would be no need to investigate if there are more effective perceptual training paradigms. Thus, the aim of the current experiment was to assess the effectiveness of standard perceptual training on a difficult real-world visual image discrimination task—grading the severity of hepatic steatosis present in ultrasound images—to determine if there is a need to develop more effective perceptual training paradigms. We spaced the training over four sessions to allow participants the time and opportunity to learn this difficult task whilst balancing fatigue and time constraints. We allowed images to repeat during training to ensure that we had sufficient stimuli, as there is evidence that repeating images is not detrimental to learning (Chen et al., 2017; Johnston et al., 2020; Sha et al., 2020).

Consistent with the literature, we hypothesised that standard perceptual training would lead to an improvement in performance, as measured by a reduction in the mean difference in error post-training (relative to the pretest). However, due to the difficult nature of the task, which requires finer discrimination than the two-choice tasks used in previous studies, we expected that participants would be unable to reduce their mean error to a benchmark level of expert performance (which we estimated from five experts that assisted with grading the stimuli). Finally, we hypothesised that learning would progress over the multiple sessions and that the average training performance (mean error) towards the end of each training session (the last 20 training trials) would improve over sessions.

### Methods

#### Participants

Participants were recruited from Prolific. A pre-screening questionnaire was used to identify participants that had normal-or-corrected to normal vision, normal colour vision, no prior training or experience in radiology, and a willingness to participate in a multiple-session experiment. We invited 100 eligible people to participate. As we expected our task would be more difficult and have a smaller effect size than the task studied by Chen et al. (2017), we recruited a substantially larger sample size (i.e. 100 instead of 25).

Data for 10 participants were excluded for non-completion of all sessions or for repeating or partially completing a session. The final sample consisted of 90 participants (*M*_age_ = 38.8 years, SD_age_ = 13.7, 45 female). Participants were compensated a total of £11.05 for completing the four sessions. To motivate performance, a bonus of £1 was awarded to the top 25% of performers.

Additionally, five experts (three consultant radiologists, one radiology fellow, and one radiology registrar) rated the stimuli. These experts were a convenience sample. The experts did not participate in the experiments. From their ratings, we also obtained an estimate of expert performance, which we used to compare the performance of our trained participants.

#### Materials

**Fig. 1:**
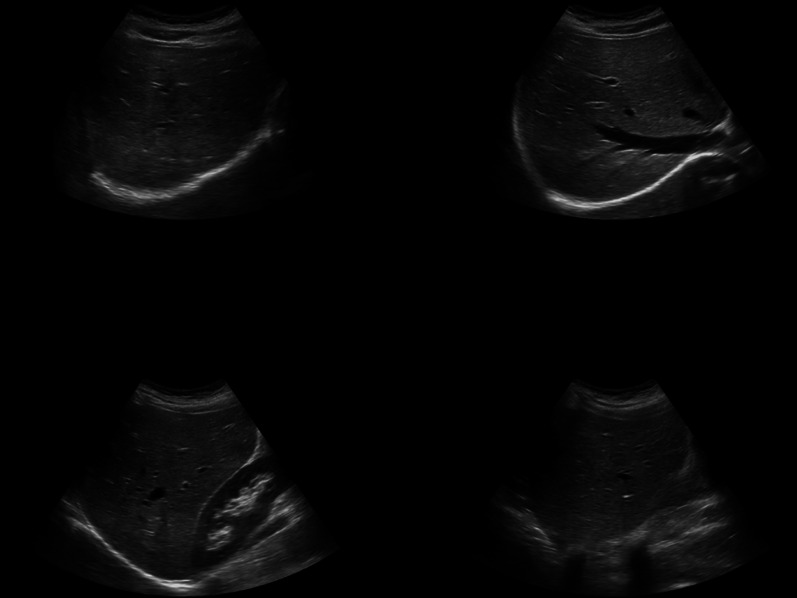
Example stimulus in Experiment 1a: collage of a liver. *Note* The collage contains two transverse and two longitudinal ultrasound views of the liver. In this example, the degree of hepatic steatosis is 2 (*Normal-mild*)

Abdominal ultrasounds of 505 unique livers were sourced from a tertiary care centre and reviewed as suitable for inclusion. Instead of using a single image of each liver, a collage image was constructed, as radiologists typically view several images when making decisions about these types of cases. Each collage contained four ultrasound views (two transverse and two longitudinal) that represented a liver (see Fig. 1 for an example).

As no objective measure is available to establish the severity of hepatic steatosis, the five experts independently graded each collage on a 7-point scale, ranging from 1 (Normal) to 7 (Severe). The grading scale was expanded so that it was more fine-grained than what is commonly used in practice, to better determine improvements in performance. For all 505 collages, the intraclass correlation coefficient estimate was 0.94, 95% CI [0.93, 0.95], which was calculated based on a mean-rating (*k* = 5), absolute-agreement, two-way random-effects model, and suggested excellent reliability (Ku & Li, 2016). For each collage, a gold standard consensus grade was determined from the average rating of the five experts. As we sought to select stimuli that were rated the most consistently by experts, collages where one or more expert’s rating deviated more than one grade from this consensus grade were excluded. The final pool of stimuli contained 386 collages. The stimuli were not equally distributed across the grades, with the majority depicting livers that were on the lower end (grades 1–3) of the scale (16%, 40%, and 11%, respectively) rather than the higher end (grades 4–7; 6%, 11%, 10%, and 6%, respectively). However, this is consistent with more severe cases occurring less frequently in practice, resulting in less suitable images of higher severity being available.

The collages were randomly split into a training (286 collages) or test set (50 collages for pretest and 50 collages for post-test), with the condition that the distribution of grades was balanced across each set. The collages were 750 pixels (width) by 562.5 pixels (height).

#### Design and procedure

The experiment was developed using jsPsych (de Leeuw, 2015) to allow for the experiment to be completed online. All participants completed the experiment on a desktop or laptop computer with a minimum browser window size of 1024 × 700 pixels.

There were four self-paced training sessions, with a pretest at the beginning of the first session and a post-test at the end of the final session. Participants had a 48-h window to complete each session, with a 24-h break between when the window for a session closed and the window for the next session opened. Therefore, depending on when in the 48-h window the sessions were completed, there was a break of at least 24 h and up to 120 h between sessions.

**Fig. 2:**
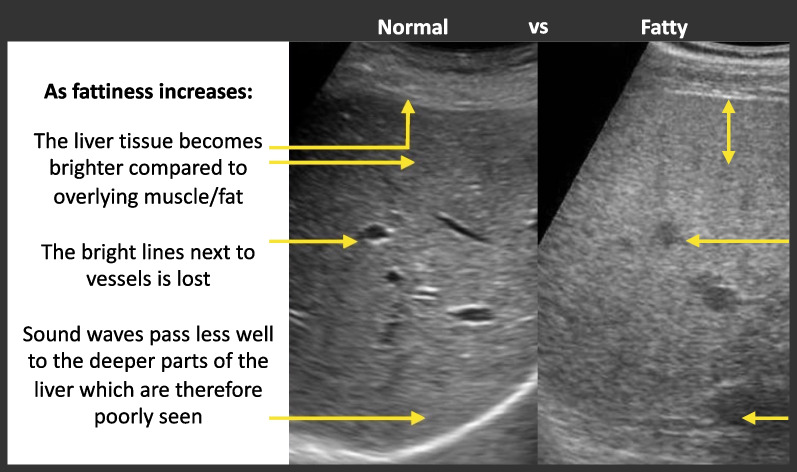
The annotated image shown in the instructions at the start of Experiments 1a and 1b

At the start of the experiment, it was explained that the task was to grade the degree of fatty liver tissue in liver ultrasound images, using the 7-point scale. The description of the task was simplified into plain language to avoid technical terms that novices may have difficulty grasping. An annotated image was shown (Fig. 2) to provide basic instruction about the type of features that differ as the fattiness increases. In addition, four individual images of livers that represented grades 1, 3, 5, and 7 were shown. This rudimentary rule-based instruction was included because it is similar to the type of instruction that radiology trainees would initially receive.

Participants then completed the pretest where they graded 50 collages, with no limits on the time taken to view the stimuli or feedback. The collages were presented sequentially in a randomised order. Responses were made via the keyboard, and a prompt was displayed underneath each collage to remind participants of the response options. No feedback was provided during this phase.

Participants then underwent four sessions of perceptual training, which was also self-paced. There were 100 training trials per session (400 in total). The collages presented during the training phase were randomly sampled with replacement from the training set. To motivate participants, points were awarded during the training phase, and these points contributed towards earning the performance bonus. Points were awarded depending on the distance from the correct answer, with a higher number of points awarded for correct responses than near-correct responses. In the training phase, after grading a collage, the correct grade was immediately presented underneath the collage with a feedback message that differed depending on how near the response was to the correct answer (i.e. “Spot on! Correct'' in green text for correct responses, “Almost” in blue text when one grade off the correct answer, “Not quite” in orange text for two grades from the correct answer, or “Incorrect” in red for responses more than two grades from the correct answer).

To encourage careful responding, ten attention check trials were included over the four sessions. On these trials, the words “attention check” were overlaid in grey text on each image in the collage and the prompt below the stimulus instructed participants on how to respond (e.g. “Please respond 1: Normal”). If an incorrect response was made, participants were reminded that it was important to pay attention to the task. Participants who failed more than one attention check on average per session (i.e. more than four out of the ten attention checks over the four sessions) were excluded from the subsequent analyses.

### Results

Due to a technical error, 1–10 trials of data were missing for five participants, so analyses were conducted on their remaining data. No participants failed the attention check criteria. The average total completion time for all sessions was 62 min.

**Fig. 3:**
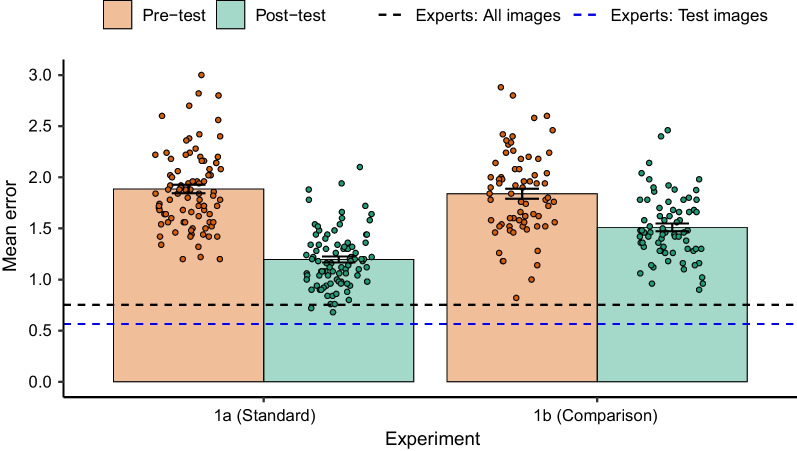
Mean error on the pretest and post-test for medically naïve participants in Experiment 1a and Experiment 1b. *Note* As the *y*-axis shows mean error (mean distance from the consensus answer), a lower value indicates better performance. The dots represent the mean error for each trained participant, and the error bars represent the standard error. The blue dashed line provides a benchmark measure of expert performance for the same images that trained participants were tested on. The black dashed line provides a comparison of expert performance for all 505 cases (i.e. before cases were excluded to select the most reliable images to use in the experiments)

As shown in Additional file 1: Fig. S1, performance on the post-test improved, with more responses closer to the consensus answer (e.g. distances 0 or 1) and fewer responses that were further (e.g. distances 5 or 6). To better quantify the overall improvement in performance, we computed the mean error for each participant on each test, which is shown in Fig. 3. The mean error represents the distance from the consensus answer, with a lower value indicating better performance. A paired-samples t test revealed that the mean error on the post-test was significantly lower than the pretest, *t*(89) = 13.68, *p* < .001, 95% CI [0.59, 0.79], *d* = 1.44.

To provide a reference point of expert performance, we first approximated the performance of our group of experts for the same collages that participants were tested on. However, we used a slightly different reference point to assess their performance, to avoid “double-dipping” the data. For each collage, each expert’s rating was assessed relative to the mean rating of the other four experts (i.e. for each expert we constructed a consensus rating using the ratings of the other four experts) and then calculated the overall mean error for the group of experts (shown in the blue dotted line in Fig. 3). As this used the same data that was used to select the reliably rated collages for use in the experiment, we also estimated the performance of the experts by repeating this procedure but for all 505 collages (shown in the black dotted line in Fig. 3). Using the results of this second more rigorous estimate of expert performance, a Welch independent samples t test found that the trained participants had significantly higher mean error than the experts, *t*(9.10) = 8.82, *p* < .001, 95% CI [0.33, 0.56], *d* = 2.14.

**Fig. 4:**
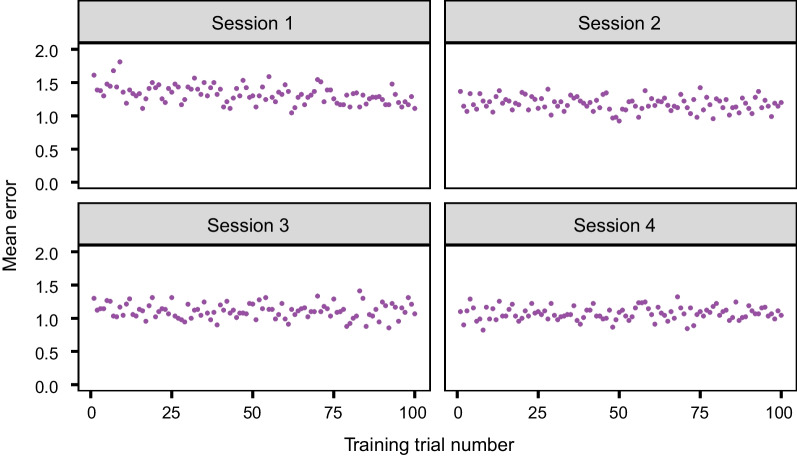
Average training performance for the Experiment 1a (standard training) sessions

Figure 4 shows the average training performance over the course of each training session. A linear model with trial number as the predictor found that the average mean error decreased significantly over the first session, *F*(1, 98) = 22.77, *p* < .001. However, this trend did not continue over the second session, *F*(1, 98) = 3.28, *p* = .073, third session, *F*(1, 98) = 0.48, *p* = .491, or fourth session, *F*(1, 98) = 0.31, *p* = .566. As we did not conduct a post-test following each training session, we approximated the learning that occurred in each session by calculating the mean error for the final 20 training trials, which is given in Table 1. A one-way ANOVA found there was a significant difference in the mean error in the final 20 trials of the four sessions, *F*(3, 267) = 17. 45, *p* < .001, η^2^_*G*_ = .08. Post hoc t tests with a Bonferroni correction revealed that the second, third, and fourth training sessions all had significantly lower mean error than the first training session (*p* < .001). All other comparisons were non-significant.

**Table 1:** Mean error (Experiment 1a) and mean difficulty level of the comparison (Experiment 1b) for the last 20 training trials of each training session

Session	Error (1a)		Difficulty level of comparison (1b)	
	*M*	SD	*M*	SD
Session 1	1.36	0.39	5.00	0.58
Session 2	1.16	0.36	5.09	0.53
Session 3	1.11	0.36	5.07	0.74
Session 4	1.07	0.34	5.13	0.60

As Experiment 1a involved standard perceptual training, mean error was used as the measure. However, as Experiment 1b involved comparison training of an adaptive nature, the measure is the mean difficulty level of the comparison

### Discussion

Consistent with our expectations and prior work, we found that perceptual training improved performance on our difficult visual discrimination task. However, unlike Chen et al. (2017), and as expected, standard perceptual training was not sufficient to train people to the level of expert performance.

When evaluating training performance, we found that meaningful improvements in performance occurred within the first training session, after which learning appeared to gradually plateau. There were no significant improvements in training performance for the later sessions. These findings are not entirely consistent with Sha et al. (2020) where there were significant improvements in learning between sessions, or with the substantial improvement over the entire training found by Johnston et al. (2020). Whilst some minor differences in methodology could account for this discrepancy (e.g. the number of images and sessions), it is plausible that our increased task difficulty limited the amount of learning that could occur with this simple perceptual training method.

## Experiment 1b

Is there a more effective training regime than the standard perceptual training approach? One alternative perceptual training method, which we refer to as *comparison training*, involves presenting several stimuli simultaneously, with the purpose of facilitating comparison. Whilst there are variations that involve passive learning (e.g. presenting stimuli with their category labels for study), we are interested in active learning where participants make judgements and receive feedback, as this kind of testing can enhance learning (Roediger & Karpicke, 2006). In active comparison training, the stimuli presented on each trial generally depict different categories (e.g. a normal and severe case) and participants need to discriminate between the stimuli (e.g. “Which image is Normal?”), and then receive immediate feedback. Whilst only a few perceptual training studies with real-world images have used comparison training (e.g. Evered et al., 2014; Searston & Tangen, 2017; Sha et al., 2020), similar techniques are successfully used in the categorisation literature (Kang & Pashler, 2012; Meagher et al., 2017). Additionally, there is some evidence that simultaneous exposure is more effective for perceptual learning than sequential exposure, in tasks with stimuli such as faces (Mundy et al., 2007) and simpler checkerboard stimuli (Mundy et al., 2009). Therefore, a perceptual training regime that involves an active comparison between simultaneously presented stimuli offers a promising way to enhance learning.

It is theorised that simultaneously presenting stimuli enhances discriminative contrast by highlighting commonalities and differences and can improve discrimination ability (Hammer et al., 2008; Kang & Pashler, 2012). This is particularly relevant when discriminating between highly similar categories (Carvalho & Goldstone, 2014). For our stimuli, those which are closer in grades (e.g. *Normal* vs *Normal-mild*) are likely to be more confusable than grades that are further apart. Therefore, the process of comparing these highly similar stimuli is expected to facilitate learning.

The aim of the current experiment is to assess if using a training approach that facilitates comparison between stimuli is effective for training medically naïve participants to grade the severity of hepatic steatosis present in ultrasound images. We hypothesised that comparison training will improve post-training performance, as measured by a reduction in mean error. Similar to Experiment 1a, we did not expect that participants would be able to reach the levels of expert performance. Although we did not find substantial benefits for multiple sessions in Experiment 1a, it is possible that comparison training could show a benefit, especially if it has the potential to teach the participant more. We therefore chose to test comparison training across four sessions, again expecting that average training performance towards the end of each training session (the last 20 training trials) would improve over sessions.

### Methods

#### Participants

The eligibility requirements to participate were the same as in Experiment 1a. However, the pre-screening questionnaire missed assessing the technical requirements of devices, so only 86 of the 100 that were invited were able to participate. Data for 15 participants were excluded for non-completion of all sessions or for repeating or partially completing a session. Thus, the final sample consisted of 71 participants (*M*_age_ = 35.5 years, *SD*_age_ = 10.8, 44 female). Participants were compensated £11.05 for completing the four sessions, with a £1 performance bonus awarded to the top 25%.

#### Materials

The stimuli were the same as in Experiment 1a. However, as the comparison task involved presented two different cases on each training trial, participants were presented with half of each collage (split vertically).

#### Design and procedure

**Fig. 5:**
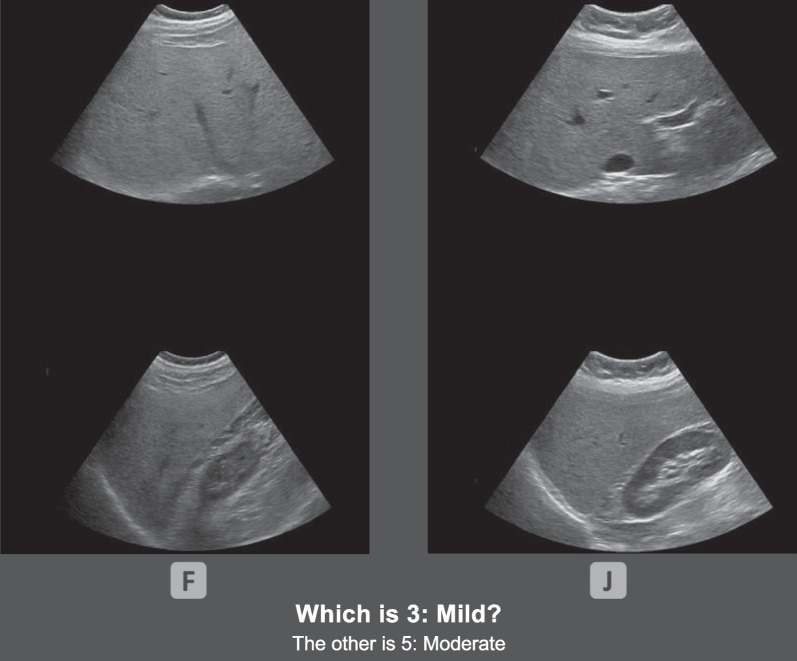
Example of a comparison training trial in Experiment 1b. *Note* The left panel contains half of a collage of a liver that is a grade 5 (Moderate) and the right panel contains half of a collage of a liver that is a grade 3 (Mild). In this example, participants were asked which panel of images were 3 (Mild) and made their response by pressing the F (left panel) or J (right panel) key. This is an example of the second most difficult comparison (level 5), where livers that are two grades apart are compared

Experiment 1b was identical to Experiment 1a except for the training task. Instead of viewing a collage of the same liver on each training trial, participants were simultaneously presented with two side-by-side collages. Each collage contained two images of the same liver, and each depicted a different grade of fatty liver disease (see Fig. 5). The decision that each collage would contain only two images was made so that on each trial, a total of four images of livers would be presented, to be consistent with Experiment 1a.

The training task differed from Experiment 1a in two other key aspects. Firstly, participants were asked to compare the two different livers and discriminate between them. At the start of each trial, participants were informed of the grade they needed to identify, along with the grade of the other liver (e.g. “Which image is 1: Normal? The other image is 7: Severe”). Participants responded according to which panel of images they believed depicted the relevant grade. Corrective feedback was immediately provided. We included ten attention check trials in total, similar to Experiment 1a, except the format was consistent with the comparison display and task.

Secondly, the training was of an adaptive nature, with the difficulty of the comparisons changing as participants progressed throughout the session. This was to balance performance and motivation. We measured the difficulty of the comparison as the distance between the grades of the livers being compared (e.g. cases that were six grades apart were the easiest and cases that were one grade apart were the most difficult). The training began with easier comparisons and followed a modified 2-up 1-down adaptive staircase procedure. After two consecutive correct responses, the difficulty of the next comparison was increased (e.g. the distance between grades decreased by one). An incorrect response stepped participants down to the previous level of difficulty (i.e. an easier comparison). Points were only awarded for correct responses, but such that higher difficulty comparisons earned more points (e.g. 1 point for the easiest comparison and 5 points for the most difficult comparison).

### Results

Due to a technical error, two participants were missing data at random for up to five trials; analyses were conducted on their remaining data. No exclusions were required for failing attention checks. Participation took an average of 86 min for the four sessions.

As shown in Fig. 3, performance on the post-test improved, with more responses closer to the consensus answer (e.g. distances 0 or 1) and fewer responses that were further. Again, we computed the average mean error for each test, which is shown in Fig. 3. A paired sample t test revealed that the mean error on the post-test was significantly lower than the pretest, *t*(70) = 6.45, *p* < .001, 95% CI [0.23, 0.43], *d* = 0.77.

We compared the mean error on the post-test of the trained participants to the same estimate of expert performance that was calculated in Experiment 1a (the more rigorous method where experts were assessed on the initial 505 collages). A Welch independent samples t test found that the trained participants had significantly higher mean error than the experts, *t*(13.38) = 13.60, *p* < .001, 95% CI [0.64, 0.88], *d* = 3.22.

**Fig. 6:**
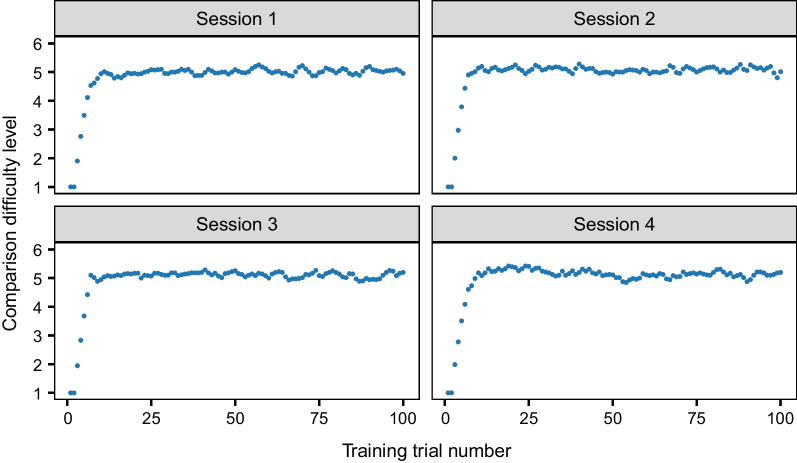
Average training performance across the Experiment 1b (comparison training) sessions. *Note* The difficulty level is represented from 1 (easiest) to 6 (hardest). The difficulty level is equivalent to the maximum grade of seven minus the distance between the grades being compared

Due to the adaptive nature of the training in Experiment 1b, we examined how the difficulty level of the comparisons progressed over the course of each training session, instead of accuracy (because accuracy is likely impacted by the difficulty of the comparison). As shown in Fig. 6, there is a rapid increase in the difficulty of the comparisons at the start of each session, as participants progress through the staircase procedure. A difficulty level of approximately five (i.e. discriminating between livers that are two grades apart) tended to be reached within the first quarter of a training session, after which performance plateaued. The same pattern occurred across each session. Table 1 displays the mean difficulty level of the comparisons for the last 20 training trials of each session. A one-way repeated measures ANOVA found no significant difference in the mean difficulty level of the comparisons for the final 20 trials between the four sessions, *F*(2.70, 189.34) = 0.93, *p* = .421, η^2^_*G*_ = .006, suggesting that the gradual improvement across sessions was not substantial.

### Discussion

We found that perceptual learning occurs when simultaneously presenting stimuli, consistent with previous work (Searston & Tangen, 2017; Sha et al., 2020). Similar to Experiment 1a, performance on the post-test improved, although did not reach the level of experts. However, we did not find a significant improvement in performance between the training sessions. A possible explanation for this unexpected finding is that after initial rapid progression, performance tended to fluctuate close to the ceiling for the remainder of a training session (as observed in Fig. 6), limiting the extent for improvement.

The improvement in performance on the post-test was significantly smaller than in Experiment 1a (see Additional file 1), despite the theorised benefits that comparison offers and although participants saw more livers overall (unique and repeated) during comparison training. One possibility for this discrepancy is that transfer of learning was limited because the training task differed from the task that participants were tested on. This is consistent with previous work which found that training on a particular task does not improve performance on an alternative task, even when the tasks involve the same stimuli (Ahissar & Hochstein, 1993). Whilst livers that depicted each of the different grades were presented during our comparison training, participants were not explicitly trained to grade the livers according to the 7-point grading scale.

Performance during the comparison training shows that participants were able to discriminate between two highly similar stimuli with a high degree of accuracy. Whilst simultaneously presenting stimuli may have facilitated comparison and improved performance on the training task, participants may have focussed on looking for similarities and differences between stimuli, rather than learning the specific perceptual features that related to each grade of hepatic steatosis. Subsequently, this could have impacted their ability to grade the stimuli according to the 7-point scale in the test.

Additionally, due to the nature of the comparison training, the distribution of livers encountered during training would not align precisely with the distribution of grades in the test set. Whereas, in Experiment 1a, the training and test set distributions were aligned, which may have facilitated learning of the underlying distribution (i.e. prevalence of each grade).

Finally, only presenting half of each collage during the comparison training limited the visual information (i.e. fewer images) that was available for each liver. Whilst this methodological choice was made to keep the total number of images presented for each liver on each training trial consistent with Experiment 1a, the reduction in visual information present for each liver may have made it more difficult to make an accurate discrimination decision.

## Experiment 2

Is it possible to improve perceptual learning further than we were able to in Experiments 1a and 1b? Recent work has found that supplementing perceptual training with annotated feedback (e.g. an arrow) that identifies the location of a target (e.g. a lesion in a mammogram), can improve learning, generalisation, (Johnston et al., 2020), and retention of learning (Frank et al., 2020). Annotations are helpful for training people to identify the location of targets (i.e. not just identifying if a target is present or not), particularly for targets that consist of more visually complex structures.

The provision of annotated feedback could even account for the substantial improvement in performance found by Chen et al. (2017), as the feedback contained arrows that identified the location of the target (hip fracture) during training. Consistent with this possibility, Johnston et al. (2020) found that although experts substantially outperformed trained novices on a more difficult task, one involving identifying whether appendicitis was present in a single axial slice in computed tomography (CT) images of abdomens, the performance gap was larger when only corrective textual feedback was provided, as opposed to more detailed annotated feedback. However, these studies have only demonstrated the effectiveness of annotations in tasks that involve judging the presence or absence of a target (i.e. a binary decision).

A similar but more explicit and detailed approach that has been used in the categorisation literature is *feature highlighting,* where feature descriptions are provided with the purpose of focussing attention on relevant features and dimensions (Meagher et al., 2021; Miyatsu et al., 2019). However, feature highlighting has only been found to be effective when the descriptions are linked to the corresponding parts of the stimulus (e.g. by circling the location; Miyatsu et al., 2019). Therefore, a training paradigm that combines annotations and descriptions of the features being identified could be particularly promising for enhancing learning, particularly for our stimuli, which require attention to multiple features.

Finally, a common approach that has been used in the education literature to facilitate the learning of complex tasks is to break the task down into a sequence of simpler steps (van Merriënboer et al., 2003). In the medical domain more specifically, sometimes a diagnosis may be reached by breaking a complex visual task down into separate categorisation decisions (Hughes & Thomas, 2021).

Therefore, the aim of the current experiment is to test two modifications to the standard perceptual training technique: (1) supplementing perceptual training with annotated feedback, and (2) breaking the training task into steps (i.e. incrementally increasing the difficulty of the task throughout the training). We used a 2 (Annotations vs No Annotations) × 2 (Steps vs No Steps) design to compare the effect of each training modification, with the *No Annotations and No Steps* condition serving as a control condition (i.e. standard perceptual training). We hypothesised that either supplementing the training with annotated feedback or modifying the training into steps will lead to more improvement on the post-test than standard perceptual training (i.e. *No Annotations and No Steps* condition). Additionally, we hypothesised that annotated feedback would improve performance more than stepped training, but that training with both modifications (*Annotations and Steps* condition) would be the most effective. We did not have a reason to expect an interaction between the Annotations and Steps conditions.

Whilst multiple training sessions may have contributed to improved performance in Experiment 1a, we believe that one training session is reasonable for initially testing our modified training approach, as we previously found that the majority of the learning occurred in the first session. We acknowledge there may be a smaller effect of standard perceptual training (i.e. *No Annotations and No Steps* condition) with one session. However, as the training modifications in the current experiment are expected to improve performance (i.e. a larger training effect) compared to standard perceptual training, one session provides an opportunity to observe the potential benefits to performance. If our modified training approach showed benefits beyond standard perceptual training, we could subsequently investigate whether multiple training sessions are beneficial.

### Methods

#### Participants

We recruited 220 participants from Amazon Mechanical Turk. As we found a large effect in Experiment 1a and because the current experiment only involved one session, so the dropout rate would likely be less, the sample size was smaller (per condition) than in Experiments 1a and 1b. All participants reported no previous experience in radiology or with ultrasound images, normal colour vision and normal-or-corrected-to-normal visual acuity. Participants were compensated $10 (USD) for completing the experiment. A bonus payment of $1 was awarded to the top 20% of participants. This experiment was pre-registered at AsPredicted: https://aspredicted.org/TK7_3PN. Data for 20 participants were excluded according to the pre-registered exclusion criteria (six for failing more than one attention check, one for missing data, eight for technical issues, and five for repeating the experiment). Our final sample (*N* = 200) included 72 females, 127 males, and 1 non-binary participant, with a mean age of 39.0 (SD = 10.5). All participants resided in the US.

#### Materials

The stimuli for the pretest and post-test were the same liver collages that were used in the respective tests in Experiment 1a and 1b. For the training stimuli, we selected a subset of 90 collages from the training set in Experiment 1a, with the stimuli selected such that the distribution of grades depicted was consistent with those in the test sets (i.e. the proportion of collages depicting each grade on the 7-point scale).

**Fig. 7:**
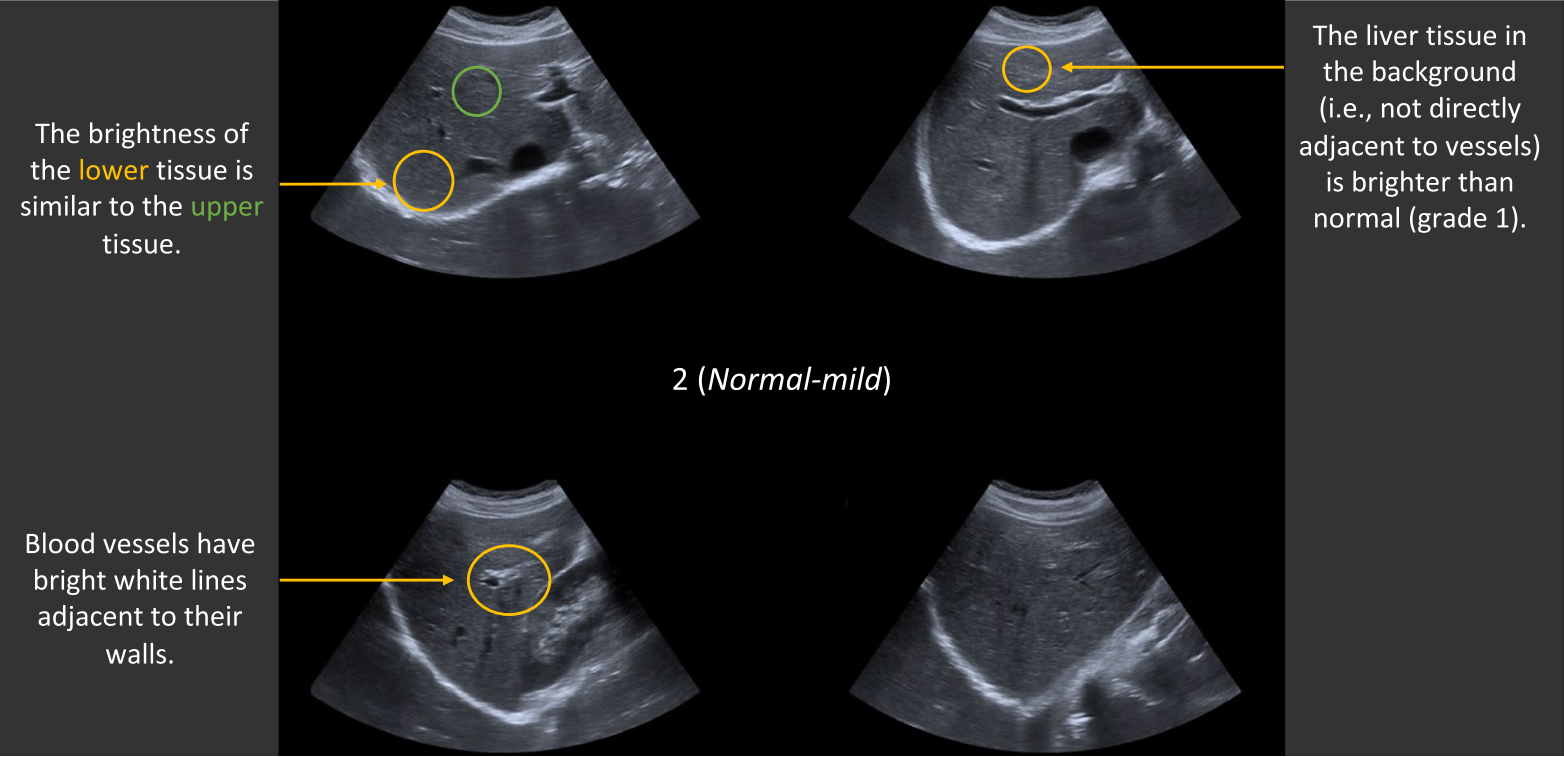
Example of annotated feedback provided in the Annotations conditions in Experiment 2. *Note* The format of the annotations was the same for the Annotations conditions, except that the number of annotated features displayed in the *Annotations and steps* condition depended on the training block (i.e. the first block only contained annotations relating to one feature, the second block contained annotations relating to two features, and the third training block contained all three features as shown in this example)

In consultation with the experts, it was determined that the training collages (in the *Annotations* conditions) would be annotated according to three key features: (a) the brightness of the background liver tissue, (b) the brightness of the white lines around the blood vessels, and (c) the difference in brightness between the lower and upper liver tissue (see Table 2 for further information). The annotations consisted of brief descriptions of the features in non-technical terms, along with arrows and circles that identified examples that were relevant for assessing each feature (see Fig. 7 for an example).

**Table 2:** The verbal descriptions used in the annotations conditions in Experiment 2

Grade	Feature 1	Feature 2	Feature 3
1	The liver tissue in the background (i.e. not directly adjacent to vessels) is not particularly bright	Blood vessels have bright white lines adjacent to their walls^b^	The brightness of the lower tissue is similar to the upper tissue^d^
2	The liver tissue in the background (i.e. not directly adjacent to vessels) is brighter than normal (grade 1)^a^	^b^	^d^
3	^a^	Blood vessels have white lines adjacent to their walls, but these are generally less bright than in grade 2	^d^
4	^a^	Some blood vessels do not have white lines adjacent to their walls. Some vessels do have white lines adjacent to their walls	^d^
5	^a^	Most blood vessels do not have white lines adjacent to their walls^c^	^d^
6	^a^	^c^	The lower tissue is slightly darker than the upper tissue
7	^a^	Almost none of the blood vessels have white lines adjacent to their walls	The lower tissue is clearly darker than the upper tissue

The first feature relates to the brightness of the background tissue, the second feature relates to the lines around vessels, and the third feature relates to the gradient between the lower and upper tissue. The same feature description could apply to more than one grade, as indicated by the letters a, b, c, and d. For example, the second description for the first feature (a) applied to grade two and above. In the two *Steps* conditions, the three features were learned incrementally over each training block (i.e. the first feature in the first training block and so on)

#### Design and procedure

Participants were randomly assigned to one of four training conditions in a 2 (Annotations vs No Annotations) × 2 (Steps vs No Steps) design. This determined whether the feedback collages during training were annotated (*Annotations* conditions) or not (*No Annotations* conditions; feedback was the same as in Experiment 1a) and whether participants were trained on each feature in a sequential fashion (*Steps* conditions) or not (*No Steps* conditions). Therefore, the *No Steps and No Annotations* condition was the same format as Experiment 1a and represents standard perceptual training and can be used as a point of comparison for the other three conditions.

The experiment was self-paced and completed online in one session. Table 3 shows the number of participants per condition and the average completion time.

**Table 3:** Number of participants and mean completion time for the conditions in Experiment 2

Condition	*N*	Mean completion time (min)
Annotations and steps	49	32
Annotations and no steps	50	28
Steps and no annotations	53	29
No steps and no annotations	48	25

As in Experiments 1a and 1b, participants were provided with instructions about the task and four example images of livers that depicted grades 1, 3, 5, and 7. Three multiple-choice questions were used to check if participants understood the task and to reduce potential data quality issues. The instructions were repeated if participants answered incorrectly. Following this, participants completed the pretest, then underwent training, and then were assessed in the post-test (the tests were the same as in Experiments 1a and 1b). Four attention check trials were included throughout the experiment to monitor data quality.

The training was split into three blocks of 30 trials, with unique collages presented in the three blocks. Within each training block the stimuli were presented in a randomised order. The same stimuli were presented in the corresponding training block across conditions (e.g. participants were presented with the same stimuli in the first block, regardless of their assigned condition).

In the *No Steps and No Annotations* condition, the training task was the same as in Experiment 1a (i.e. grading each collage on the 7-point scale without any annotations). However, in the two *Steps* conditions, participants were instructed to use a particular feature (or features) to make their judgement in each training block. A brief multiple-choice understanding check was used to assess if participants understood the instructions in each block (i.e. the feature/s and task) and the instructions were summarised if participants answered incorrectly. In the first training block, participants were instructed to consider the first feature (i.e. the brightness of the background liver tissue). As this feature alone is generally only helpful for distinguishing a grade 1 (Normal) from higher severity grades, the task was simplified; participants graded each collage as either a grade 1 or more than a grade 1. In the second training block, participants were instructed to additionally use the second feature (i.e. the brightness of the white lines around the blood vessels). Together, both features are generally useful for distinguishing between grades 1 and 4, but not between grades that are higher than 4. Accordingly, task difficulty increased, and participants graded each collage as either a grade 1, 2, 3, 4, or more than 4. Finally, in the third training block, participants were informed of the third feature (i.e. the difference in brightness between the lower and upper liver tissue) and instructed to consider all three features when grading the stimuli. Therefore, the task in the third (and final) training block required participants to grade each collage using the full 7-point grading scale (i.e. the same task they were tested on).

In the *Annotations and Steps* condition, participants were only presented with annotated feedback that related to the feature (or features) they had learned about up to that current block (e.g. only one feature in the first block). However, in the *Annotations and No Steps* condition, participants were presented with annotated feedback that related to all three features.

### Results

**Fig. 8:**
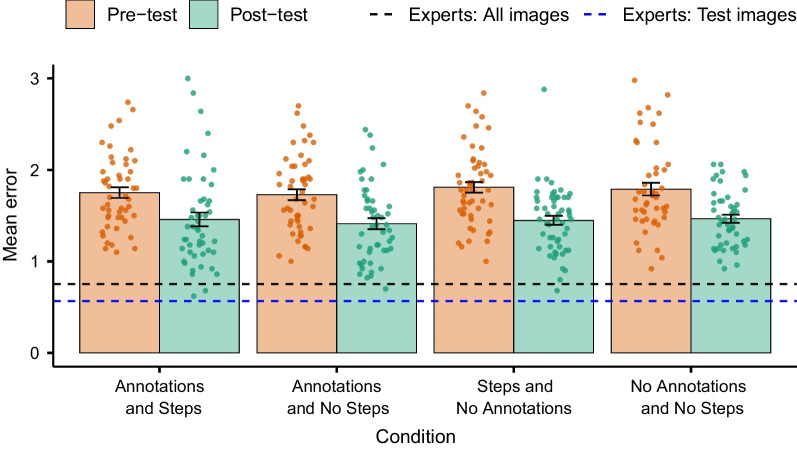
Mean error on the pretest and post-test for each training condition in Experiment 2. *Note* The dots represent the individual data (mean error for each participant), and the error bars represent the standard error. Whilst the analysis conducted was on the mean difference between the pretest and post-test, the data for each test are displayed for ease of comparison

Additional file 1: Fig. S2 shows the distribution of responses on the pretest and post-test for each condition. The trend is similar across conditions and with Experiment 1a and 1b. Before proceeding with our main analysis, we collapsed our data across all conditions to assess if there was an overall training effect. A paired-samples t test found that the reduction in mean error from pretest to post-test was significant, *t*(199) = − 9.80, *p* < .001, 95% CI [− 0.39, − 0.26], *d* = 0.69. Therefore, we proceeded with our main analysis, to compare the training conditions. We conducted a 2 (Annotations) × 2 (Steps) between-participants ANOVA, with the mean difference in error between the pretest and the post-test as the dependent measure. Figure 8 shows that this difference was similar across conditions. Contrary to our expectations, there was no significant main effect of *Annotations*, *F*(1, 196) = 0.56, *p* = .454, or *Steps*, *F*(1, 196) = 0.06, *p* = .809. Additionally, there was no significant interaction between *Annotations* and *Steps*, *F*(1, 196) = 0.21, *p* = .648.

Whilst we had not pre-registered this analysis, we compared the mean error on the post-test (collapsed across all four training conditions since we found no significant difference in performance) to the estimate of expert performance on the original 505 collages. An independent samples Welch t test found that the trained participants had a significantly higher mean error (*M* = 1.45, SD = 0.41) than the experts (*M* = 0.75, SD = 0.09), *t*(8.99) = 13.88, *p* < .001, 95% CI [0.58, 0.81], *d* = 2.34.

### Discussion

In contrast to our expectations, providing detailed annotated feedback did not aid learning. Whilst this finding is inconsistent with previous work (Frank et al., 2020; Johnston et al., 2020; Miyatsu et al., 2019), differences in the nature of the task and our stimuli could account for this. For example, in Frank et al. (2020), participants only needed to search for a single target (e.g. a lesion or grouped microcalcification in a mammogram image), and in Johnston et al. (2020), participants only needed to attend to a specific location (e.g. identifying the location of the appendix and deciding if it appeared normal), so there was likely a clear benefit to drawing attention to the location of the target or appendix. Conversely, in our study, participants needed to attend to various locations and multiple cues to make their judgement. Additionally, our annotations may have not been as helpful because the relevant features varied along a continuum (e.g. the brightness of the lines around blood vessels) as opposed to features that are clearly either present or absent. Relatedly, the effectiveness of feature highlighting can depend on how diagnostic the features are for distinguishing between confusable categories (Meagher et al., 2021). It could be that the features that we focussed on did not have sufficiently high diagnostic power.

Contrary to our expectations, breaking the training into discrete steps also did not improve performance. Focussing on learning the features in a sequential fashion may not be helpful if the features do not have sufficient independent diagnostic power. For instance, some categories are extremely difficult to master with explicit verbal instruction, particularly if multiple features need to be considered in a holistic fashion to make an accurate judgement (Hughes & Thomas, 2021). If the three features that we trained participants on are only useful when considered together, then attempting to learn them sequentially would be ineffective.

## General discussion

Our work makes several contributions to the growing literature on perceptual training. In Experiment 1a, we investigated to what extent we could use perceptual training to improve performance on a complex, real-world visual discrimination task. We used a very simple training regime: participants were shown collages of liver ultrasound images and graded the severity of the hepatic steatosis for each, then received immediate feedback as to the actual severity, as determined by the collective judgement of a group of experts. We demonstrated that this simple perceptual training method led to a significant improvement in performance, although participants were unable to improve to the level of experts.

In Experiment 1b, we replicated these findings using a different training regime, which facilitated comparison between different grades of stimuli. Participants were simultaneously presented with two half-collages that each represented a different liver. They were told the grade of the hepatic steatosis for both livers but were not told which grade applied to each liver. Participants judged which grade applied to each liver and received immediate feedback. After two consecutively correct responses, the comparison task was made more difficult by reducing the difference between the grades of hepatic steatosis in the two half-collages. Conversely, following an incorrect response, the task was made easier. In this way, participants were always presented with judgements near the limit of their ability. Performance improved rapidly within a training session but did not significantly improve across subsequent training sessions (perhaps due to the high level of performance that was attained early on). Perhaps longer training sessions for both Experiment 1a and 1b would have resulted in improved learning between each session and across the entire course of the training.

Following our inability to improve the performance of participants in Experiments 1a and 1b to the level of experts, we changed our approach for Experiment 2. Previously, the expert radiologists had indicated that there were three features that they particularly focussed on. When presented with the results from our first two experiments, these radiologists suggested training participants on these features in a sequential fashion. Additionally, a subsequent literature search suggested that explicitly annotating the features might increase learning. Therefore, we investigated both factors using a crossed 2 × 2 design. Despite less training than in Experiments 1a and 1b, participants were able to improve their performance on the post-test. However, to our surprise, neither the stepwise training nor the annotations significantly enhanced performance beyond standard perceptual training, even when combined. These results reinforce our findings from Experiments 1a and 1b; although perceptual training can rapidly improve the performance of participants on this task, participants were unable to reach similar levels of performance as the experts.

It is possible that the methods we used to estimate expert performance are biased and do not reflect their true performance on this task. Our first method estimated the experts’ performance from the 100 test collages and was likely an overestimate, as only the reliably rated collages had been selected for use in the experiment. However, our second method, where performance was estimated from all 505 collages, is arguably more rigorous and potentially even biased against experts, as it evaluated performance on a set of collages that were likely harder to classify. An unbiased measure of expert performance likely lies somewhere between our two estimates. Regardless, participants were not able to achieve the level of performance of experts even when the second method was used to estimate expert performance.

Another consideration is the practical usefulness of the training, despite participants not reaching expert performance as measured in our studies. As the grading scale used in our experiments was more fine-grained than what is commonly used in practice, the level of skill achieved by the training could still be considered useful in a practical sense. Therefore, we collapsed the 7-point grading scale to a less fine-grained grading scale (4-point scale) that is more commonly used in practice and repeated our analysis (reported in the Additional file 1). However, even when using the less fine-grained grading scale, participants did not achieve the level of performance of experts.

The level of identification skill that participants were able to achieve in our training compared to prior work may have been impacted by differences in the task. For instance, the tasks that Chen et al. (2017) and Johnston et al. (2020) studied only required a two-choice identification of a single image (e.g. fracture or no fracture), where chance performance was 50%. In contrast, our task was more difficult as it involved a more fine-tuned discrimination that required participants to attend to multiple features and locations across several views of a liver (i.e. the collage) to make their decision. It is possible that a short amount of perceptual training can be effective at training novices to the level of experts for two-choice identification tasks that involve the clear presence or absence of certain features, but not for more fine-grained tasks that require sensitivity to multiple features, even when annotated feedback is provided. Although the novices in Johnston et al.’s (2020) study did not reach expert performance, this could be because they were only trained to make each diagnosis based on a single CT image (in practice experts make use of multiple images) and the appendix being more difficult to identify on a single CT image than a femur in an X-ray image (as in Chen et al., 2017).

The task studied by Chen et al. (2017) was a true perceptual task. To identify whether a fracture existed, participants needed to learn what a normal head of the femur looks like and then determine if the femur in the test image differed sufficiently from normal. In other words, participants were comparing what they saw to their visual representation of how ‘normal’ looks. As the location of fracture (if present) was similar in each case, participants likely learned to rapidly direct their attention to the relevant area. Crucially, they could do this task without needing to understand how the X-ray image was created or why the head of femurs appear the way they do. Such information was not required for their diagnosis.

For our study, making a diagnosis was more complex. On further questioning the experts that provided the expert ratings for our stimuli, it was apparent that they were basing their diagnosis on an understanding of how liver ultrasound images are created along with knowledge of how the structure of a normal liver looks like and how hepatic steatosis affects the appearance of this structure. Instead of just making a perceptual judgement, the experts performed a series of deductions, based on their extensive background knowledge. To complicate matters further, the diagnosis process often differed from image to image. Not all the cues that were relevant in one image would necessarily be relevant in a second image. For example, for one image, the first observation an expert may make is that the ultrasound gain when acquiring the image was too high. They were able to make this determination based on their knowledge of what liver blood vessels should look like on an ultrasound. The determination that the gain was too high influenced what cues they subsequently considered. What cues need to be considered and even what the cues mean therefore varied from image to image. A successful diagnosis needs to utilise both an understanding of how the image was formed as well as how hepatic steatosis can affect the underlying structure of the liver and how this, in turn, affects how the liver appears in ultrasounds. In diagnosing the images, the experts were drawing upon a wealth of detailed, domain-specific knowledge.

When designing Experiment 2, we were aware that experts were using background knowledge to inform their judgements. Although we couldn’t hope to teach our participants the background medical knowledge that the experts had, we wondered to what degree we could approximate the expert decision-making process without it. This is what prompted our discussions with experts to determine what strategies they were using. However, the process and strategies that experts engage in when making a diagnosis may not be entirely represented in their explanation of the process they undertake (Feldon, 2007). This is because it can be difficult for experts to articulate implicit knowledge that they rely on (Roads et al., 2016). Additionally, the features or cues that experts and novices perceive to be useful for diagnosis can differ (Robson et al., 2020). Therefore, detailed annotations that are based on the cues that experts verbally describe as relevant for making a diagnosis may not translate well when training novices. An interesting alternative training approach that may overcome such challenges suggests using *attentional highlighting*, whereby novices are trained to follow the gaze patterns that experts use whilst completing the task (Roads et al., 2016). It is unclear whether this approach would offer any additional training benefits for tasks that seem to rely heavily on domain-specific knowledge but is a potential avenue for future research.

Finally, it is important to acknowledge that expertise is typically acquired after many years and experience with a wealth of exemplars. Therefore, it is not surprising that we were unable to train people to achieve expert performance in such a short amount of training. The features (or rules) that we trained participants on in Experiment 2 may have been unhelpful because the information that they provide is too broad or abstract. Specifically, these rules do not provide information about the instantiations of features—the varying perceptual manifestation of features (Brooks & Hannah, 2006). Gaining a vast amount of experience with different exemplars aids in learning how features are instantiated. Therefore, future research could investigate whether expert levels of performance could be achieved in this task by simply providing a longer amount of training.

In conclusion, taken together, our experiments indicate some limits of perceptual training. Although we found that perceptual training can lead to rapid improvements in performance, even for a more difficult medical image discrimination task, our participants were not able to achieve expert levels of performance. It is possible that our task requires extensive domain-specific knowledge to reliably interpret the images, or alternatively that the amount of training was not sufficient. Future work will be needed to better identify the types of tasks where perceptual training is likely to be most useful, but the current work offers a useful starting point. The current work also emphasises the importance of distinguishing between the rate of the increase in performance and the total increase in performance. Whilst it has been repeatedly shown that perceptual training typically results in an initial rapid increase in performance, it is equally important to determine what level of performance can be achieved by perceptual training, relative to the level of performance achieved by experts. Historically, the perceptual training literature has tended to neglect the latter point.

## Supplementary Information

**Additional file 1. **Supplementary analyses and figures for Experiments 1a, 1b, and 2.

## Data Availability

The data and stimuli are available on the Open Science Framework: https://osf.io/dsvge/.
