# Supplementary material for: Evaluating the effectiveness of different perceptual training methods in a difficult visual discrimination task with ultrasound images
Source: Cogn Res Princ Implic. 2023 Mar 20;8:19. doi: 10.1186/s41235-023-00467-0 (PMC10027970; doi:10.1186/s41235-023-00467-0)
Supplement: Supplementary file 1 — Additional file 1. Supplementary analyses and figures for Experiments 1a, 1b, and 2. [file 41235_2023_467_MOESM1_ESM.docx]

**Evaluating the Effectiveness of Different Perceptual Training Methods in a Difficult Visual Discrimination Task with Ultrasound Images**

**Supplementary Materials**

Jessica E. Marris, Andrew Perfors, David Mitchell, Wayland Wang, Mark W. McCusker, Timothy John Haynes Lovell, Robert N. Gibson, Frank Gaillard, and Piers D. L. Howe

*Corresponding author: Jessica Marris (jess.e.marris@gmail.com)*

**Distribution of Responses**

To provide a more detailed breakdown of changes in performance on the pre-test and post-test, the percentage of responses for each possible distance from the consensus answer (derived from the experts) are shown in Figure S1 for Experiments 1a and 1b, and in Figure S2 for Experiment 2. Across all experiments, there were more responses that were closer to the consensus answer (mean distance of 0) on the post-test (green bars) compared to the pre-test (orange bars), indicating that some learning occurred.

**Comparing Performance in Experiment 1a and 1b**

The mean difference in performance between the pre-test and post-test was calculated for Experiments 1a and 1b. An independent samples Welch t-test found that the mean difference in performance (between the pre-test and post-test) was significantly larger for participants that underwent standard perceptual training (*M* = 0.69, *SD* = 0.48) than participants that underwent comparison training (*M* = 0.33, *SD* = 0.43), *t*(156.33) = 5.04 *p* < .001, 95% CI [0.22, 0.50], *d* = 0.79. Whilst these findings suggest that the standard perceptual training was more effective, this interpretation is made with caution as participants were not randomly allocated to training conditions (the data was collected sequentially) and the comparison training task differed to the task that participants were tested on.

**Recoding Stimuli and Collapsing the Grading Scale**

In our experiments we used a more fine-tuned (7-point) grading scale than what is commonly used in practice. We explored how collapsing the grades to a scale that is used in practice (4-point scale) would impact on our findings. We recoded grade 1 to 0 (normal cases), grades 2 and 3 to 1, grades 4 and 5 to 2, and grades 6 and 7 to 3.

We recoded the grades that the experts provided for the 505 collages and calculated the consensus grade. For all 505 collages, the intraclass correlation coefficient estimate was .92, 95% CI [.90, .93], which was calculated based on a mean-rating (*k* = 5), absolute-agreement, 2-way random-effects model, and suggested excellent reliability (Ku & Li, 2016). We subsequently recoded the responses that participants made in Experiments 1a and 1b, since the training was longer (and had a larger effect) in these experiments than in Experiment 2.

The percentage of responses for each possible distance from the consensus answer are shown in Figure S3 and the mean error is shown in Figure S4. Paired samples t-tests revealed significantly higher post-training performance for participants that underwent standard perceptual training, *t*(89) = 13.02, *p* < .001, 95% CI [0.28, 0.38], *d* = 1.37, and comparison training, *t*(70) = 6.23, *p* < .001, 95% CI [0.11, 0.21], *d* = 0.74. Independent samples Welch t-tests found that the participants that underwent standard perceptual training had significantly higher mean error than the experts, *t*(7.38) = 5.58, *p* < .001, 95% CI [0.10, 0.24], *d* = 1.50, as did participants that underwent comparison training, *t*(9.11) = 10.27, *p* < .001, 95% CI [0.25, 0.40], *d* = 2.76.

**Figure S1**

*Percentage of Responses for Each Distance From the Consensus Answer in the Pre-test and Post-test for Experiment 1a and Experiment 1b*


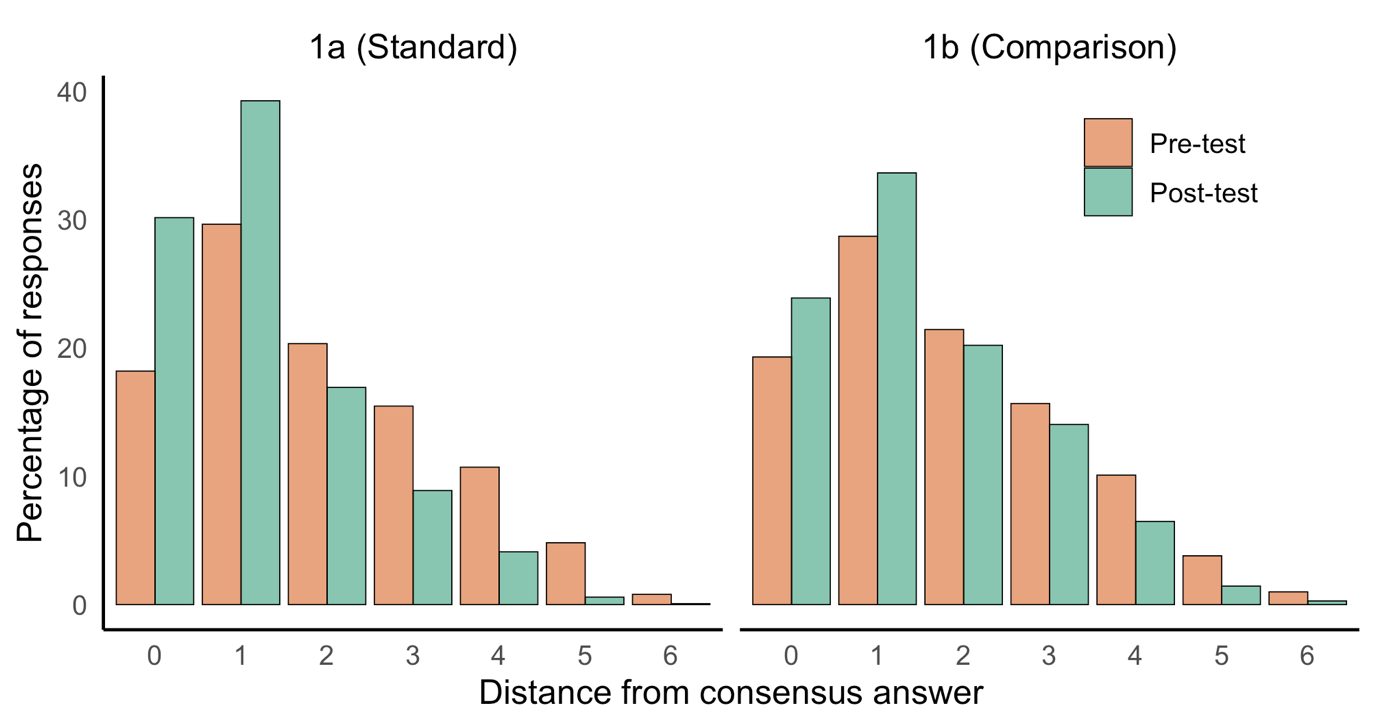


*Note*. The data displayed is from the participants that underwent perceptual training. A distance of 0 is equivalent to a correct response. In both Experiment 1a and 1b, there was a higher percentage of correct or near-correct answers in the post-test (green bars) than in the pre-test (orange bars), indicating some learning occurred.

**Figure S2**

*Percentage of Responses for Each Possible Distance From the Consensus Answer in the Pre-test and Post-test for Experiment 2*


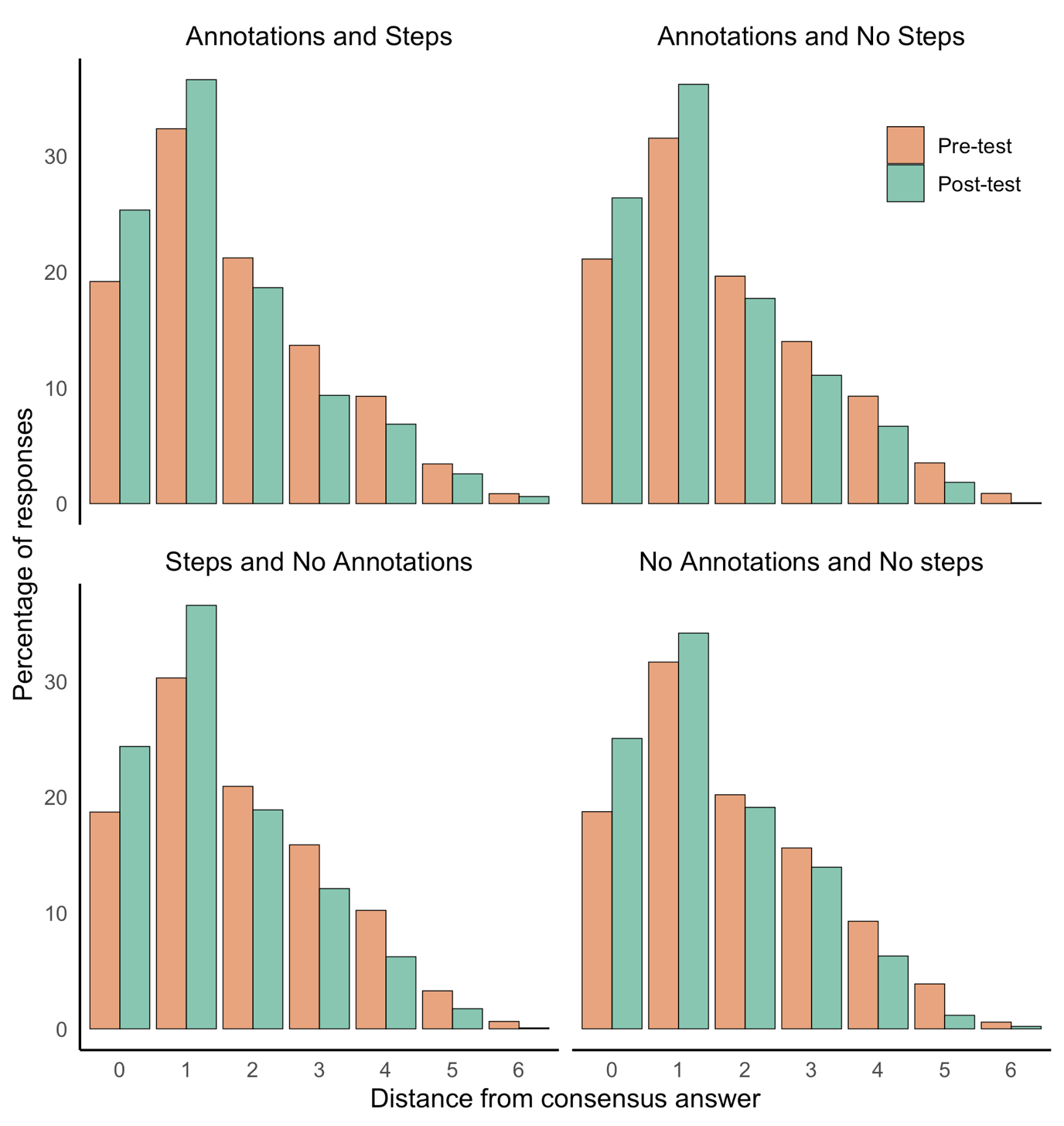


*Note*. There was a higher percentage of correct (i.e., distance of 0) or near-correct responses on the post-test (green bars) compared to the pre-test (orange bars), indicating some learning occurred.

**Figure S3**

*Percentage of Responses for Each Distance From the Consensus Answer in the Pre-test and Post-test (Recoded Data) in Experiment 1a and Experiment 1b*


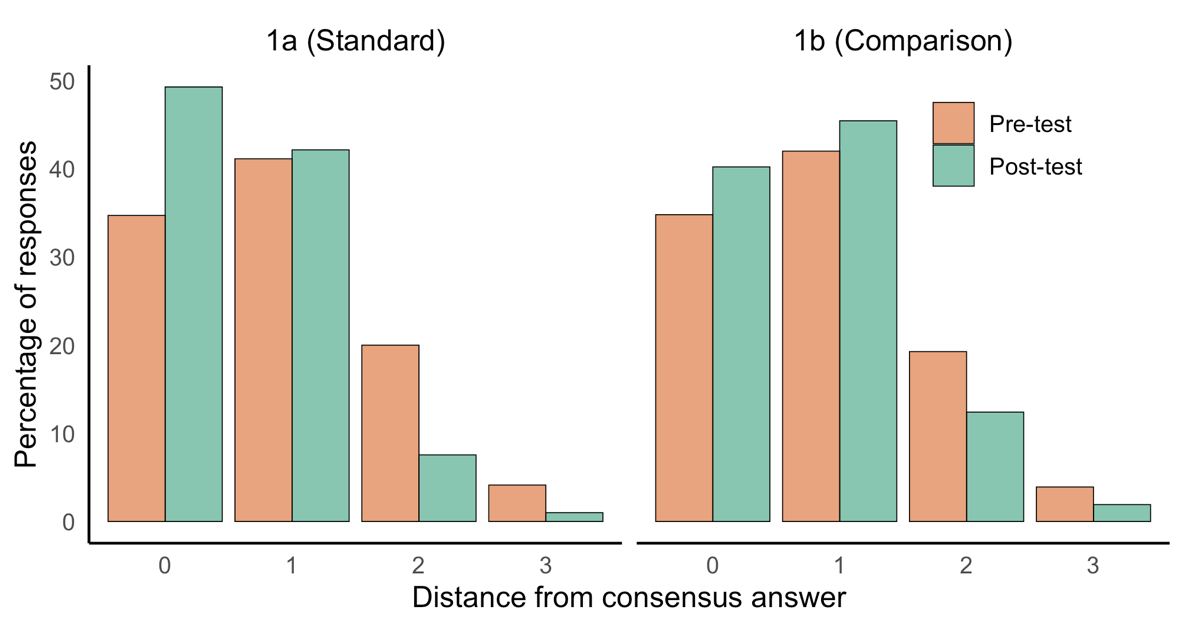


*Note*. The data was recoded to collapse the 7-point scale used in the experiments into a less fine-grained scale (4-point) that is similar to what is used in practice. The data displayed is from the participants that underwent perceptual training. A distance of 0 is equivalent to a correct response (consistent with the consensus answer). In both Experiment 1a and 1b, there was a higher percentage of correct or near-correct answers in the post-test (green bars) than in the pre-test (orange bars), indicating some learning occurred.

**Figure S4**

*Mean Error on the Pre-test and Post-test (Recoded Data) for Medically Naïve Participants in Experiment 1a and Experiment 1b*

**
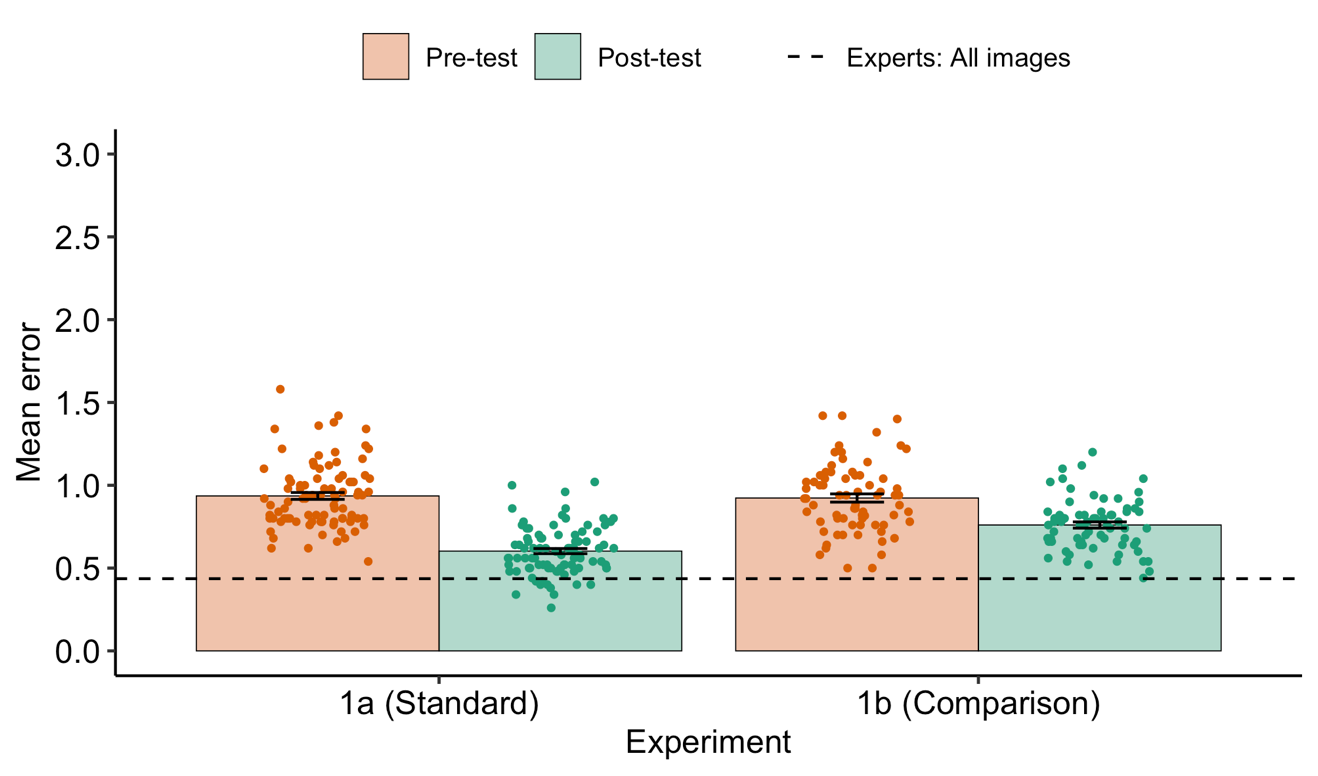
**

*Note.* The data was recoded to collapse the 7-point scale used in the experiments into a less fine-grained scale (4-point) that is similar to what is used in practice. As the y-axis shows mean error (mean distance from the consensus answer), a lower value indicates better performance. The dots represent the mean error for each trained participant and the error bars represent the standard error. The black dashed line provides a comparison of expert performance for all 505 cases (i.e., before cases were excluded to select the most reliable images to use in the experiments).
